# Supplementary material for: Improving Formation Conditions and Properties of hBN Nanosheets Through BaF2-assisted Polymer Derived Ceramics (PDCs) Technique
Source: Nanomaterials (Basel). 2020 Feb 29;10(3):443. doi: 10.3390/nano10030443 (PMC7152994; doi:10.3390/nano10030443)
Supplement: Supplementary file 1 [file nanomaterials-10-00443-s001.pdf]

nanomaterials

MDPI

Type of the Paper (Article-Supplementary Information)

Improving Formation Conditions and Properties of *h*BN Nanosheets Through BaF_2-assisted Polymer Derived Ceramics (PDCs) Technique

Boitumelo J. Matsoso [1], Victor Vuillet-a-Ciles [1], Laurence Bois [1], Bérangère Toury [1] and Catherine Journet [1,*]

[1] Laboratoire des Multimatériaux et Interfaces, UMR CNRS 5615, Univ Lyon, Université Claude Bernard Lyon 1, F-69622 Villeurbanne Cedex-France; boijo.matsoso@gmail.com (B.J.M.), victor.vuillet-a-ciles@univ-lyon1.fr (V.V.C.), laurence.bois@univ-lyon1.fr (L.B.), berangere.toury@univ-lyon1.fr (B.T.)

* Correspondence: catherine.journet@univ-lyon1.fr; Tel.: +33-4724-335-64

1. Methods

From a reaction of ammonium sulphate and sodium borohydride in tetraglyme, a pure monomer of borazine was synthesized, after which it was polymerized at 55 °C inside a pressure-sealed system under argon for 5 d [1], in order to generate polyborazylene (PBN) [2]–[5]. Under an inert argon atmosphere inside a glovebox, PBN was later mixed with 5 wt% of lithium nitride (Li_3N) and varying amounts (0-10 wt%) of barium fluoride (BaF_2). After homogenizing the pre-ceramic mixture by stirring for 10 min, this mixture was then heated to 200 °C in an alumina crucible and kept for 1 h to give a solid-state polymer [6], [7], [8]. Finally, *h*BN nanosheets were synthesized by annealing the stabilized pre-ceramic mixture for 1h at 1200 °C (1 °C/min) under inert nitrogen (N_2) atmosphere.

2. Results

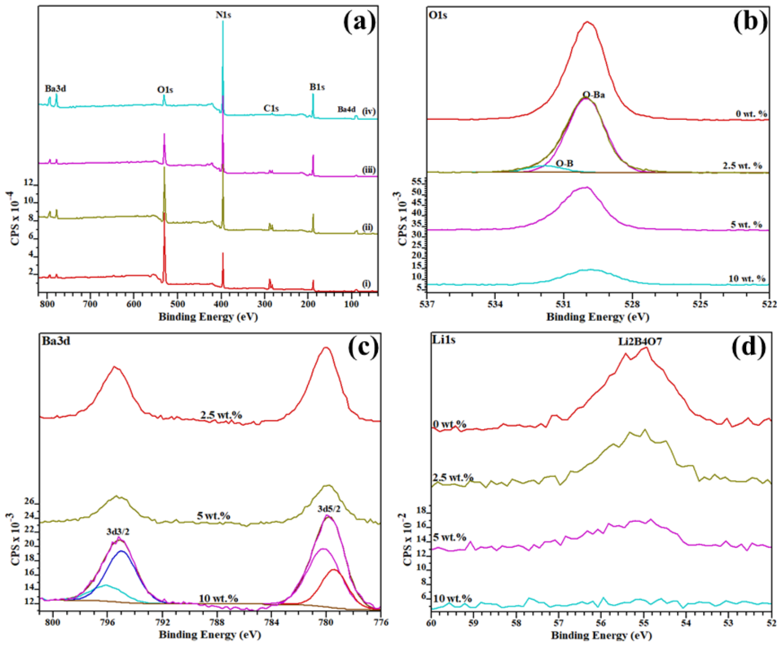

Figure S1. (a) Survey spectra of the sample before sputtering with Ar^+ ions, as well as high resolution of (b) O1s, (c) Ba3d, and (d) Li1s.

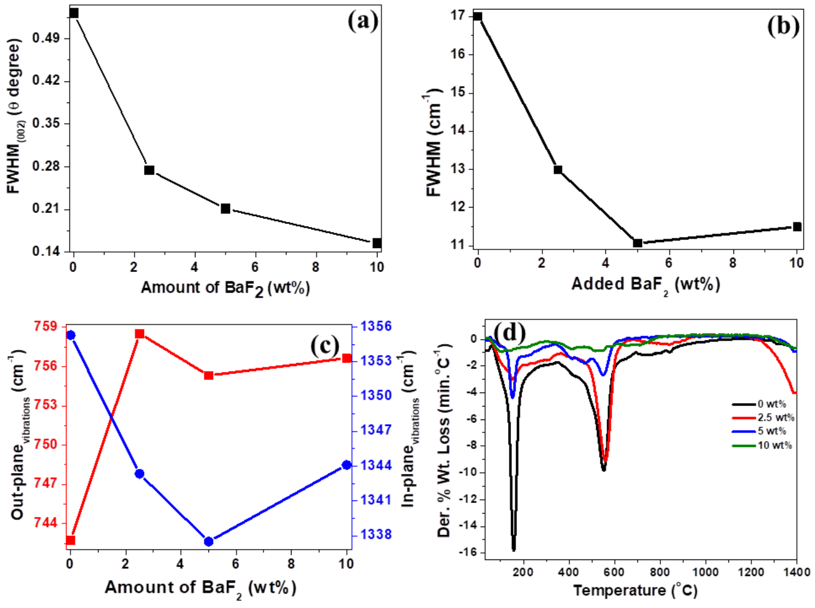

Figure S2. Relation of increasing addition of BaF_2 to (a) FWHM of (002) XRD peak (b) Raman peak FWHM, and (c) FTIR out- and in-plane vibrational modes. (d) DTG plot of the as-synthesized samples.

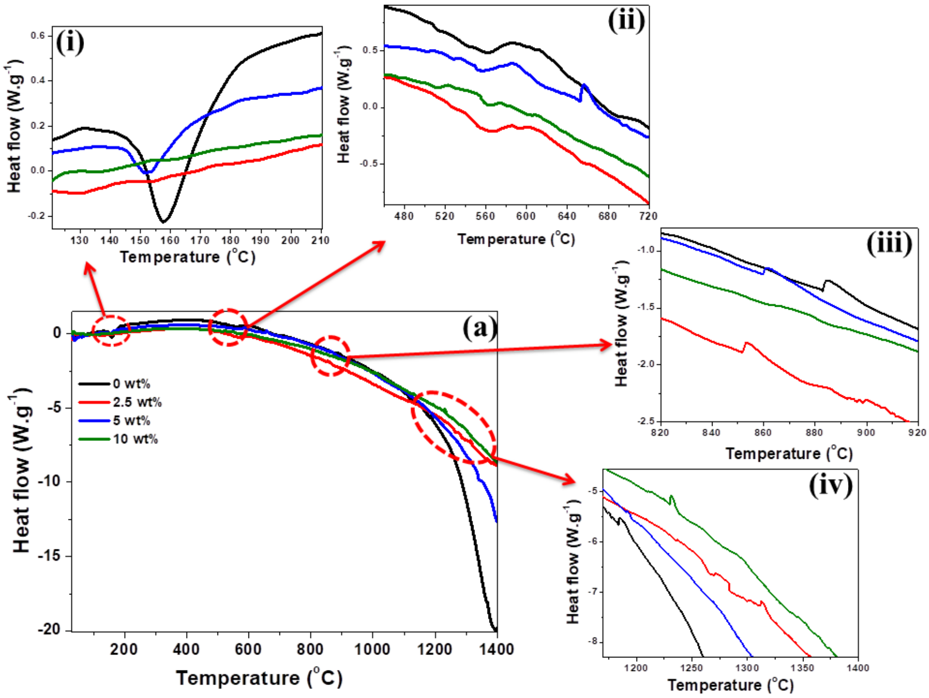

Figure S3. (a) DSC plots for the as-synthesized samples: (i)-(iv) Regions of exothermic and endothermic reaction during sample decomposition.

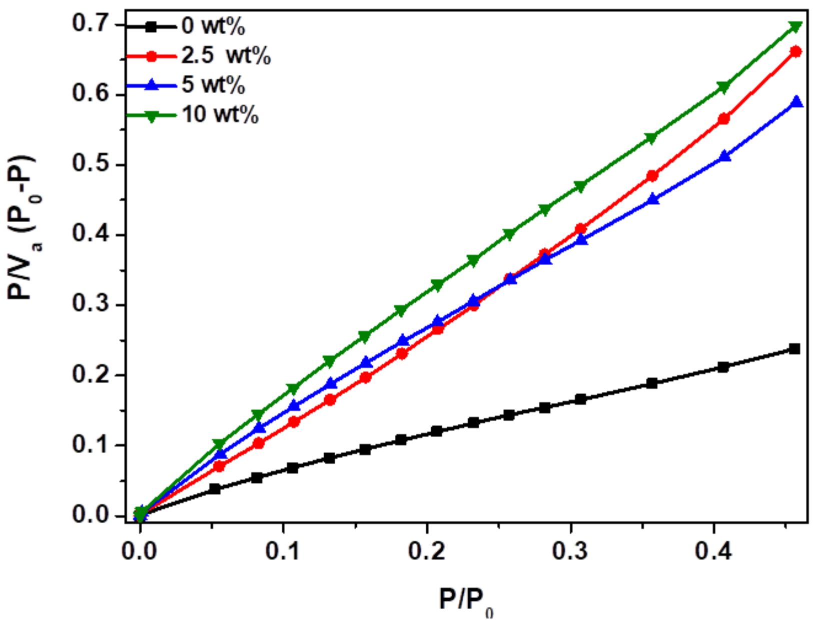

Figure S4. Multi-point BET plots in the relative pressure range 0.05 < P/P0 < 0.30 for the as-synthesized samples.

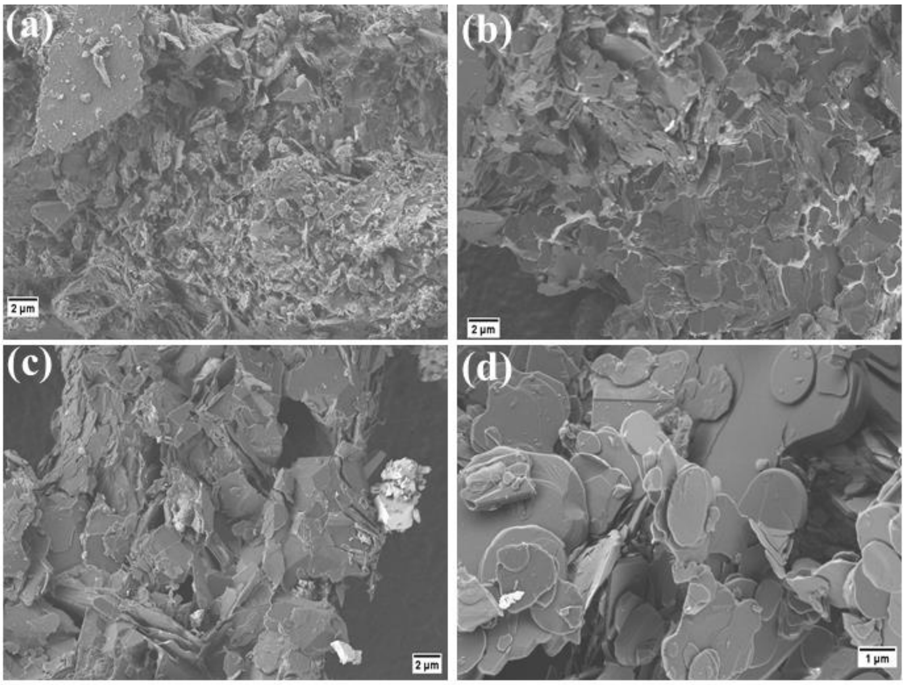

Figure S5. SEM micrographs of as-synthesized BNNS samples annealed at 1200 °C, after addition of 5 wt% Li_3N and (a) 0, (b) 2.5 and (c) 10 wt% BaF_2. (d) SEM micrograph of nanoplatelets of commercial *h*BN (99%, Alfa Aesar).

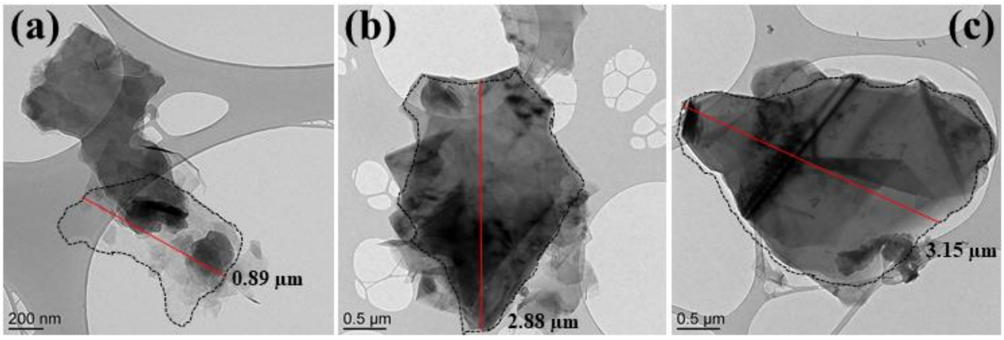

Figure S6. Low magnification TEM images of BNNS samples annealed at 1200 °C, after addition of 5 wt% Li_3N and (a) 0, (b) 2.5 and (c) 10 wt% BaF_2.

Table S1. TGA parameters for the as-synthesized *h*BN samples

Sample (wt% BaF_2)	Decomposition Temp (°C)		On-set Temp (°C)	↑$Mass_{temp}$ (°C)
0	156.6	547.3	74.2	-
2.5	154.3	559.7	66.8	934.9
5	153.5	548.3	82.9	663.9
10	102.5	529.5	68.8	788.1

Table S2. Textual parameters of the as-synthesized *h*BN samples

Sample (wt% BaF_2)	BET S.A. (m^{-2}/g)	Pore Diameter (nm)	Pore Volume (cm^{-3}/g)
0	8.7	14.8	0.03
2.5	3.5	25.0	0.02
5	3.6	17.9	0.02
10	2.9	21.0	0.02
hBN_{comm}	6.7	15.5	0.03

References

1. T. Wideman and L. G. Sneddon. Convenient Procedures for the Laboratory Preparation of Borazine. *Inorg. Chem.*, vol. 34, no. 4, pp. 1002–1003, 1995.
2. S. Bernard and P. Miele. Polymer-derived boron nitride: A review on the chemistry, shaping and ceramic conversion of borazine derivatives. *Materials (Basel).*, vol. 7, no. 11, pp. 7436–7459, 2014.
3. C. Gervais *et al.* Chemically Derived BN Ceramics: Extensive 11B and 15N Solid-State NMR Study of a Preceramic Polyborazilene. *Chem. Mater.*, vol. 13, no. 5, pp. 1700–1707, May 2001.
4. D. Cornu, P. Miele, B. Toury, B. Bonnetot, H. Mongeot, and J. Bouix. Boron nitride matrices and coatings from boryl borazine molecular precursors. *J. Mater. Chem.*, vol. 9, no. 10, pp. 2605–2610, 1999.
5. H. Termoss, B. Toury, A. Brioude, J. Dazord, J. Le Brusq, and P. Miele. High purity boron nitride thin films prepared by the PDCs route. *Surf. Coatings Technol.*, vol. 201, no. 18, pp. 7822–7828, 2007.
6. S. Yuan, B. Toury, C. Journet, and A. Brioude. Synthesis of hexagonal boron nitride graphene-like few layers. *Nanoscale*, vol. 6, no. 14, pp. 7838–7841, 2014.
7. S. Yuan *et al.* Low-Temperature Synthesis of Highly Crystallized Hexagonal Boron Nitride Sheets with Li_3N as Additive Agent. *Eur. J. Inorg. Chem.*, vol. 2014, no. 32, pp. 5507–5513, Nov. 2014.
8. B. N. Feigelson, R. M. Frazier, and M. Twigg. III-Nitride crystal growth from nitride-salt solution. *J. Cryst. Growth*, vol. 305, no. 2 SPEC. ISS., pp. 399–402, 2007.
